# The Nervous Power of Science Versus the Blood-Life of Nature. A Fragment of a Natural Physiology, Pathology, &c., of the Nervous System

**Published:** 1846-07

**Authors:** 


### Art. XXII.

*Die Nervenkraft im Sinne der Wissenschaft gegenüber dem Blutleben in der Natur. Rudiment einer naturgemässern Physiologie, Pathologie und Therapie des Nervensystems.* Von Dr. Carl Jos. Heidler, &c. —*Braunschweig*, 1845.

*The Nervous Power of Science versus the Blood-life of Nature. A Fragment of a natural Physiology, Pathology, &c. of the Nervous System.* By Dr. C. J. Heidler, Imperial Austrian Councillor, Senior Official Physician to the Baths at Marienbad, &c.—*Brunswick*, 1845. 8vo, pp. 392.

The regular current of sciential progress is broken at intervals by some queer-shaped rock or uncouth apparition which rises or "crops up" above the ripples. The disturbance thus created is sometimes considerable; there is much chafing, and roaring, and muddiness, before the obstruction is fairly removed. Usually, however, the disturbance is trifling; just so much as an uncouth fish might make when lifting its head above the waves. Not long ago one book was published to demonstrate that the theory of the circulation of the blood was an ingenious philosophical fiction—no more: and another, to set forth a mystery about the blood and muscle; in which the professional public was requested to take notice that blood and muscle were the all in all, and nerve comparatively nothing. Well, now here we have, in Dr. Heidler's book, a still more uncouth fish popping its queer-looking head above the rippling current of physiological and pathological research; resolved, apparently, to give a new direction to the stream, or, failing in that, to make at least a stir by its queerness. The blood, according to Dr. Heidler, is the first, the last, the supreme in the organism. The blood receives impressions from the external world, and is the medium of communication between these and the nerves. The blood is the immediate and active stimulant of all organs and tissues. There is no nervous power; there are no nervous diseases. The former is a fiction, the latter are dependent upon morbid states of the circulation; in short, nervous diseases, so called, are caused by congestion of the capillaries.

The object (to be more specific) of Dr. Heidler's dissertation, is to show that the practical study of the physiological, pathological, and therapeutical relations of congestions, either with or without "orgasmus," and of spontaneous or primary, and of secondary or consensual hemorrhages, is the most useful, intelligible, and certain means of advancing physiology, pathology, and therapy. A change in the quantity or (less certainly) in the quality of the blood and in the stimulation by the blood of tissues and organs, is the immediate or proximate general cause of all animal sensations, from the first or lowest degree of common sensation to those of the highest degree, and which involve pleasure or pain. The numerous modifications which sensations exhibit are dependent on modifications of the blood. These differ in degree, kind, and locality, according to the organ in which they take place. The nervous system is a machinery for communication only; an apparatus for "animal perception; in animals an animal addition,—in man a humano-animal addition to the vegetative and inorganic vital processes in both. Its functions are simply to be a medium for communicating vibrations, oscillations, or undulations developed by concussions. It perceives and directs.

Certain "introductory remarks" are made up of a variety of gossip. We have references to previous works; references to private criticisms made by persons to whom Dr. Heidler submitted the manuscript of his book; statements of what he did intend, of what he now intends, of what he thinks on this, that, and the other. This preliminary flourish accomplished, he sets himself seriously to work. He first pulls down the theory antagonistic to his own. His first effort is to prove a negative,—that the so-called nervous principle or power does not exist. He examines the evidence in its favour under the five different heads of anatomy, physiology, pathology, therapy, and metaphysics. This he does after a crafty method; he collects the notions of various modern writers as to the nature of the soul, the nervous principle, perception, sensation, volition, and elicits a variety of palpable contradictions. Opposing views on these subjects are also cleverly mixed up with ideas of a more rational character, in such a way that we cannot sufficiently admire the author's ingenuity. At the same time, he has a knack of asking puzzling questions, and one really begins to feel a regard for the shrewd and clever author. He appears to advantage as a good puller-down. When, however, we come to examine into his own theory, and into his powers of building up, we find a woful declension. The proofs of his proposition,—that the nerves are merely an apparatus for the conduction of oscillations, that the blood receives the impressions of these oscillations and transmits them to the nerves, and that these oscillations do occur, consist simply in the vaguest analogies. It would be a useless tax upon the patience of our readers to set forth these arguments at length, and to demonstrate their absurdity. We will, however, present them with a specimen of the foggy notions in which Dr. Heidler indulges. It is well known that in certain neuralgic cases there is great sensibility to currents of air, to sounds, to light, to heat; and that paroxysms of pain are readily induced by these stimuli. Dr. Heidler thus discourses on this matter, and develops his theory:

"Lastly, it must not here be left out of consideration, that both kinds of occasions of increase of pain through light and heat, are amongst the obvious supports of my proposition, that pain is caused through the blood. Such a support to this proposition is—*a*, the increase of pain in consequence of the increase of blood in the affected parts, occasioned by the bio-chemical affinities of the blood for light and heat; and, *b*, the increase of pain in consequence of the increase of blood, the result of the vibrations in the atmosphere, caused by light and warmth. It is easily understood that the blood will be for the moment increased in quantity by the vibration communicated to the air, and the blood be morbidly congested in the morbidly painful tissue.

"Is not this communicated vibration plainly no other than a diminutive and retarded likeness of the pulse of the blood—of this other larger and slower modification of my law of periodicity of all-life in creation—the most fundamental and general of all natural laws? (vide §§ 15, 16, &c.) Do we not see very acute pain increased with each stroke of the pulse? And what is this vibratory motion of the blood (and of its vessels, and all solids)—this image of the pulse on a reduced scale—what else are they both than a small ebb and flow of the blood in a closed space? What else are they than a removal of the aggregated parts into another, and the one into the other, and a compression and heaping up there from whence they are removed; through each pulse-stroke, as well as through each vibratory impulse? What else is both, than that relatively excessive (arterial) congestion in the small vessels above [quoted] from the programme to the 'new theory of pain' (better, theory of sensation)?

Further: truly what else is this increase of pain, effected by means of these pulse-strokes and vibratory impulses, than the twice-diminished similitude of all periodic efforts of nature; of the curative as well as the destructive exacerbation and remission—of the paroxysm and of the intermission of disease,"&c.&c. (p. 218.)

This extract is a fair example of our author's lucubrations, and the theory which he proposes as a substitute for that which he has so diligently destroyed. It has been our fate to grope through many cross fogs of the German intellect, but we never yet were so absolutely at a loss for one guiding ray as during our pilgrimage through Dr. Heidler's fog. One thing is evident; Dr. Dickson (the chrono-thermal sage) will be down upon him for plagiarism. There is mention made of "my law of periodicity of all-life in creation."

With regard to the pathological and therapeutical views of Dr. Heidler, we have only to remark that they are simple enough, and old enough. Congestion of the abdominal viscera, either induced by the suppression of hemorrhoidal discharges, or accompanied by them, is the proximate cause of neuralgic and spasmodic affections. The treatment recommended is—*to keep the bowels open.*

